# A Thermal Imaging Camera as a Diagnostic Tool to Study the Effects of Occlusal Splints on the Elimination of Masticatory Muscle Tension

**DOI:** 10.3390/dj13070313

**Published:** 2025-07-11

**Authors:** Danuta Lietz-Kijak, Adam Andrzej Garstka, Lidia Szczucka, Roman Ardan, Monika Brzózka-Garstka, Piotr Skomro, Camillo D’Arcangelo

**Affiliations:** 1 Department of Propaedeutic, Physical Diagnostics and Dental Physiotherapy, Faculty of Medicine and Dentistry, Pomeranian Medical University, 70-204 Szczecin, Poland; danuta.lietzkijak@gmail.com (D.L.-K.); adamgarstka87@gmail.com (A.A.G.); lkozlowska@op.pl (L.S.); 2 Department of Economics, Faculty of Economic Sciences, Koszalin University of Technology, 75-343 Koszalin, Poland; rardan@interia.pl; 3 Department of Physiotherapy, University of Physical Education, 61-871 Poznań, Poland; on.brzozka@gmail.com; 4 Reparto di Odontoiatria Restaurativa e Endodonzia, Università Degli Studi “G. D’Annunzio”, 66100 Chieti, Italy; camillo.darcangelo@gmail.com

**Keywords:** infrared thermography, thermal imaging camera, medical infrared thermography, occlusal splints, temporomandibular disorders, masticatory muscle tension, muscle tone, myofascial pain, jaw pain

## Abstract

Medical Infrared Thermography (MIT) is a safe, non-invasive technique for assessing temperature changes on the skin’s surface that may reflect pathological processes in the underlying tissues. In temporomandibular joint disorders (TMDs), which are often associated with reduced mobility and muscle overactivity, tissue metabolism and blood flow may be diminished, resulting in localized hypothermia. Aim: The purpose of this study was to evaluate muscle tone in the masseter, suprahyoid, and sternocleidomastoid muscles following the application of two types of occlusal splints, a Michigan splint and a double repositioning splint, based on temperature changes recorded using a Fluke Ti401 PRO thermal imaging camera. Materials and Methods: Sixty dental students diagnosed with TMDs were enrolled in this study. After applying the inclusion and exclusion criteria, participants were randomly assigned to one of two groups. Group M received a Michigan splint, while group D was treated with a double repositioning splint. Results: The type of occlusal splint influenced both temperature distribution and muscle tone. In the double repositioning splint group, temperature decreased by approximately 0.8 °C between T1 and T3, whereas in the Michigan splint group, temperature increased by approximately 0.7 °C over the same period. Conclusions: Occlusal splint design has a measurable impact on temperature distribution and muscle activity. The double repositioning splint appears to be more effective in promoting short-term muscle relaxation and may provide relief for patients experiencing muscular or myofascial TMD symptoms.

## 1. Introduction

Thermal imaging cameras are widely used in medicine as non-invasive diagnostic tools. These devices detect infrared radiation and convert it into color maps that represent surface temperature distributions across the body or in a specific region [1,2,3]. Medical Infrared Thermography (MIT) is particularly useful for identifying variations in skin temperature that may indicate pathological processes in the underlying tissues [4]. Injuries often lead to changes in blood flow and vascular integrity, resulting in elevated skin temperature (hyperthermia). Conversely, joint disorders—especially those associated with reduced mobility and muscle activity—can cause diminished tissue metabolism and blood flow, leading to localized hypothermia [5]. MIT has applications across various medical disciplines, including vascular medicine, neurology, orthopedics, oncology, sports medicine, and dentistry [6,7,8,9,10,11,12]. Studies have also explored its use in patients with leprosy and dermatological conditions [13,14], as well as in the evaluation of athletes using an infrared thermal camera [15,16,17]. The human body can be compared to a complex, precise machine composed of interconnected systems that enable diverse biomechanical functions. These connections involve structures such as muscles, ligaments, fascia, joints, bones, nerves, and blood vessels. Each element is a link in a functional chain, and dysfunction in one can influence the performance of others [18,19]. In the field of temporomandibular disorders (TMDs), thermography offers a non-contact, real-time method for evaluating muscle activity and inflammation, with notable advantages over traditional methods such as electromyography (EMG) and palpation [20,21]. Unlike EMG, which requires surface electrodes and is sensitive to motion artifacts, or palpation, which is inherently subjective, thermography provides an objective visualization of physiological responses related to muscle tone and blood flow. TMDs are often characterized by increased tension in the masticatory muscles, pain in the temporomandibular joints, limited mandibular movement, headaches, and additional symptoms such as joint noises or tinnitus [22]. Effective treatment typically requires interdisciplinary cooperation between dentists, physiotherapists, and other healthcare providers [23,24]. According to the Diagnostic Criteria for Temporomandibular Disorders (DC/TMDs), muscle-origin pain is classified as either myalgia or myofascial pain syndrome (MPS) [25]. Myalgia is usually localized and triggered by movement or palpation, whereas MPS is associated with referred pain and palpable trigger points within the muscles [26,27,28,29,30,31,32,33,34]. Among the conservative treatment options for TMD, occlusal splints are widely used. The Michigan splint is commonly prescribed for patients with muscular dysfunction or bruxism [35,36]. It redistributes occlusal forces and reduces mechanical stress on the temporomandibular joint, an effect considered to be a key biomechanical mechanism of action [37]. While MIT has previously been used to assess inflammation and muscle function in TMDs, no studies to date have directly compared the thermal effects of different occlusal splint types. This study is the first to evaluate and compare temperature-based muscle responses following the application of a Michigan splint and a double repositioning splint.

**Hypothesis:**  *We initially hypothesized (null hypothesis) that both splint types would produce similar thermal responses over time. Alternatively, we proposed that temperature changes and thus muscle tone would differ significantly depending on the splint type applied.*

Clinical relevance: Identifying temperature-based differences between splint types may help clinicians select the most effective therapeutic strategy for achieving muscle relaxation in patients with myogenous or myofascial TMD.

**Aim of the study:** The aim of this study was to investigate muscle tone in the masseter, suprahyoid, and sternocleidomastoid muscles after the application of a Michigan splint and a double repositioning splint, as assessed by temperature changes measured using a Fluke Ti401 PRO thermal imaging camera.

## 2. Material and Methods

This study included 60 dental students aged 20–25 years who reported symptoms of temporomandibular disorders (TMDs). All participants were recruited from the Faculty of Dentistry and screened based on clearly defined inclusion and exclusion criteria (Table 1). Eligible students were randomly assigned to one of two treatment groups using sealed, opaque envelopes prepared in advance by an independent researcher who was not involved in participant assessment or treatment. This procedure ensured proper allocation concealment.

- Group M received a Michigan splint;
- Group D received a double repositioning splint.

This study protocol was reviewed and approved by the Bioethics Committee of the Pomeranian Medical University in Szczecin, Poland (KB-006/89/2024). Written informed consent was obtained from all participants prior to enrollment.

***Occlusal Splints***

**Figure 1:**
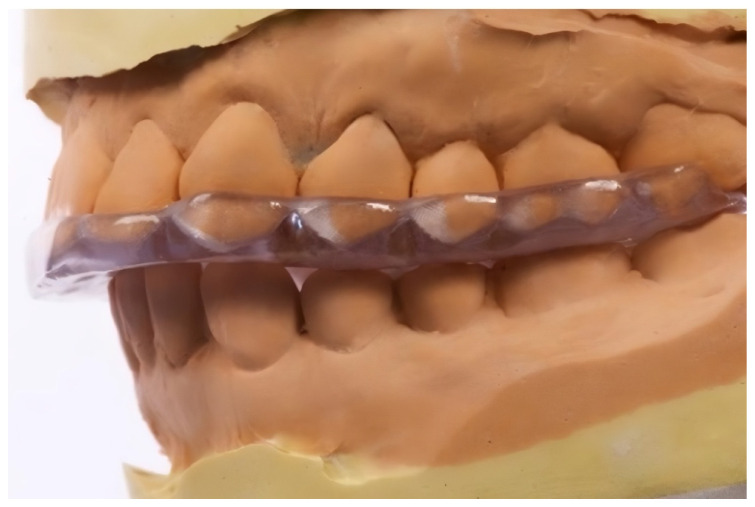
The Michigan splint, used in the study group M.

**Michigan Splint (Group M) (Figure 1):**

This splint was fabricated from hard acrylic to cover the maxillary arch.

It was designed for the following purposes:

- Achieve centric relation;
- Provide freedom in the centric (0.5–1.0 mm flat surface);
- Establish canine guidance beginning ~1 mm from the centric;
- Eliminate incisal guidance from centric occlusion;
- Allow the ideal seating of the condyles in the articular fossae.

**Figure 2:**
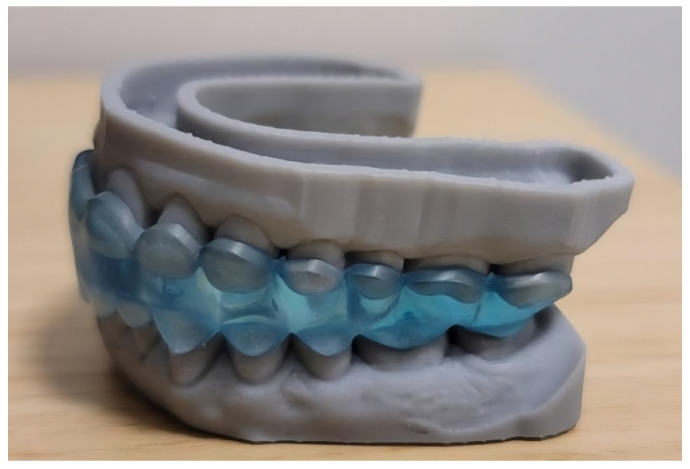
The double repositioning splint used in study group D.

**Double Repositioning Splint (Group D) (Figure 2):**

This custom-fabricated splint consists of separate upper and lower components designed to stabilize the mandible and prevent undesired mandibular movement during wear. The splint was manufactured using Digital Light Processing (DLP) technology, a high-precision 3D printing method that cures the photopolymer resin layer by layer using a digital light projector. This technique ensures excellent accuracy, surface quality, and fit, which are critical in intraoral applications. For optimal clinical performance, the splint was printed using Ortho Flex resin by NextDent, a material that fulfills the following two essential requirements:

- Full biocompatibility, certified by the manufacturer, ensuring safety for intraoral use;
- Elasticity and durability, which allow for the repeated insertion and removal of the splint while maintaining mechanical strength under functional loading conditions.

The mandibular position for each patient was determined individually by the treating dentist through a functional assessment. Patients were instructed to wear the splint primarily at night for a period of 1 to 3 months. To support reproducibility, Figure 1 and Figure 2 present visual schematics of both splint types, illustrating their functional zones and occlusal contact surfaces.

***Thermographic Measurements***

Each participant underwent thermal imaging of the masseter, suprahyoid, and sternocleidomastoid muscles at three time points:

- **T1**—Baseline (at rest, before splint application);
- **T2**—Immediately after splint placement;
- **T3**—30 min after splint placement.

**Figure 3:**
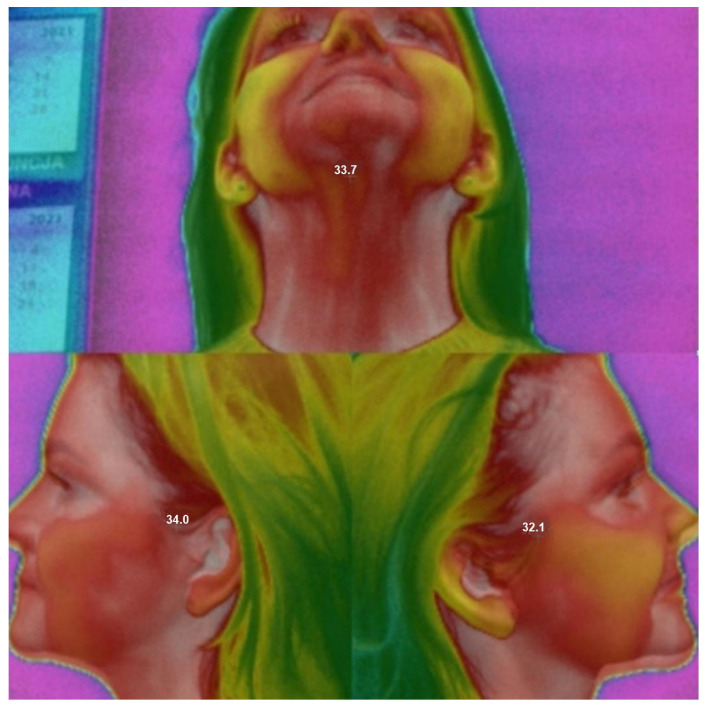
Thermography of the masseter, suprahyoid, and sternocleidomastoid muscles. The photo shows numbers indicating the temperature at the time of measurement.

All thermographic measurements (T1 to T3) were conducted during the same clinical session under standardized conditions. Participants wore their assigned occlusal splint continuously between T2 and T3 for a duration of 30 min, without removing it during that period. Measurements were taken in a temperature-controlled room by a trained operator using a Fluke Ti401 PRO thermal imaging camera (resolution: 640 × 480 pixels; thermal sensitivity: ≤0.075 °C at 30 °C; accuracy: ±2% at 25 °C). The operator was blinded to the group allocation to minimize measurement bias. Each thermographic image was analyzed to calculate the mean surface temperature in the central belly of each muscle, as indicated in Figure 3. The following assumptions were made:

- Active muscles generate more heat, which is transmitted via vascular and fascial structures.
- Thermal imaging can detect these changes with high accuracy.
- Overloaded or tense muscles are expected to exhibit higher surface temperatures compared to relaxed muscles.

### 2.1. Sample Size

The sample size was calculated using the following formula:

$$n=\frac{2{\left(z_{1-\frac{\alpha }{2}}+z_{1-\beta }\right)}^{2}}{d^{2}}$$

where

*n*—the number of patients in each group;
α—the probability of a type I error (false positive);
β—the probability of a type 2 error (false negative);
*d* = ($\mu_{1}-\mu_{2}$)/σ—Cohen’s effect size;
$\mu_{1},\mu_{2}$—sample mean in both groups;
σ—standard deviation.

The essential difference in the averages is taken to be equal to 0.6 grade. The estimation of the standard deviation is based on previous clinical studies [20]. The standard deviation (SD) is taken to be approximately 0.8. Therefore, based on these data, we assumed d ≈ 0.75. For α = 0.05 and β = 0.2, the formula gives *n* ≈ 28.

Based on these calculations and considering the approximate value of the SD, a sample size of a minimum 30 patients was considered.

### 2.2. Statistical Analysis

To test the significance of temperature changes at successive time points and throughout the observation period, Welch’s *t*-test was used separately in each group. To investigate the temperature differences between the groups at three time points and temperature changes at successive time points and throughout the observation period, Welch’s *t*-tests for two samples with Bonferroni correction were used. Results were considered statistically significant at *p* < 0.05. Statistical analyses were carried out using the R software package, version 4.1.2 (The R Foundation for Statistical Computing, Wirtschaftsuniversität Wien, Vienna, Austria).

## 3. Results

Temperature measurements were obtained from each participant at three anatomical sites:

- N—masseter muscle;
- OSP—suprahyoid muscles;
- OSL—sternocleidomastoid muscles.

Data were collected at three time points:

- T1—baseline (before splint placement);
- T2—immediately after splint placement;
- T3—30 min after splint placement.

Measurements focused on the central belly of the muscle, particularly in areas identified during clinical palpation as tense or containing trigger points.

Descriptive statistics for each muscle group across both treatment arms (Michigan splint—M; double repositioning splint—D) are presented in Table 2. The results include mean temperature values, standard deviations, and confidence intervals.

At baseline (T1), no statistically significant differences in muscle temperature were found between the groups (*p* = 0.604 for N, 0.723 for OSP, 0.179 for OSL), confirming the comparability at the start of the study.

In the double repositioning splint group (D), a progressive temperature decrease was observed at all three anatomical sites from T1 to T3. In contrast, in the Michigan splint group (M), temperature values increased over time across all measured locations.

**Figure 4:**
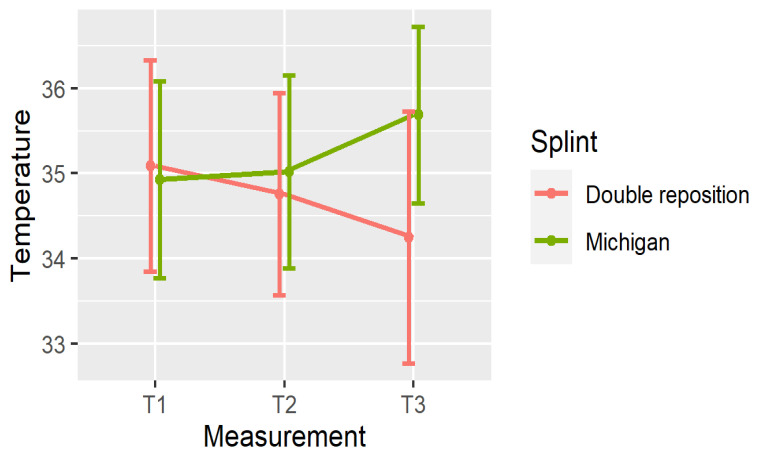
Mean ± SD of temperature in N at three time points.

**Figure 5:**
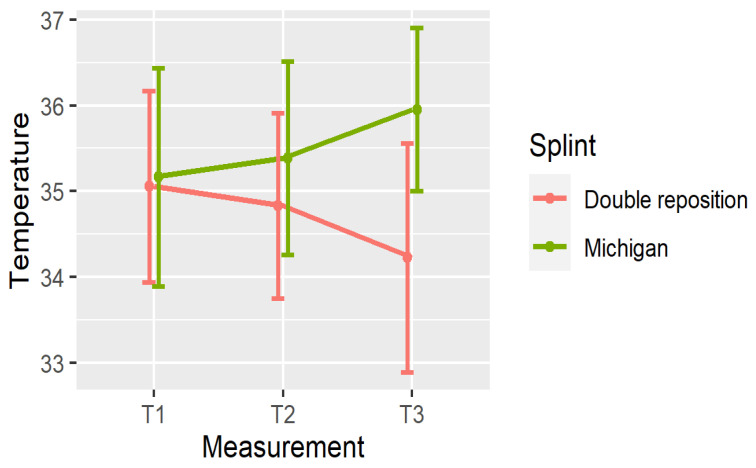
Mean ± SD of temperature in OSP at three time points.

**Figure 6:**
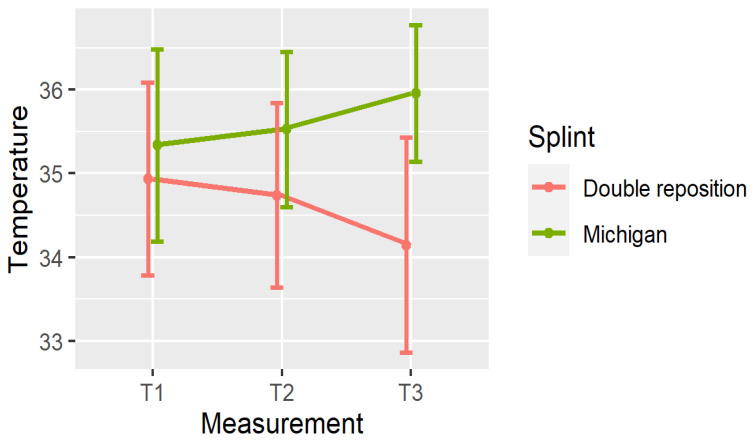
Mean ± SD of temperature in OSL at three time points.

Figure 4, Figure 5 and Figure 6 illustrate the mean temperatures with the standard deviations for each muscle at the three time points. These visualizations help highlight the inverse trends in thermal responses between the two groups.

From a clinical standpoint, the observed decrease in surface temperature in the D group may indicate a reduction in muscle hyperactivity and tension. Conversely, the increase in temperature in the M group could suggest continued or increasing muscular activation.

## 4. Changes in Temperature

To evaluate the effect of the occlusal splints over time, temperature changes between the three time points were calculated for each muscle group. The variables represent the following:

- Dnij—change in temperature in the masseter muscle from time point *Ti* to *Tj*;
- DOSPij—change in the suprahyoid muscles;
- DOSLij—change in the sternocleidomastoid muscles.

**Figure 7:**
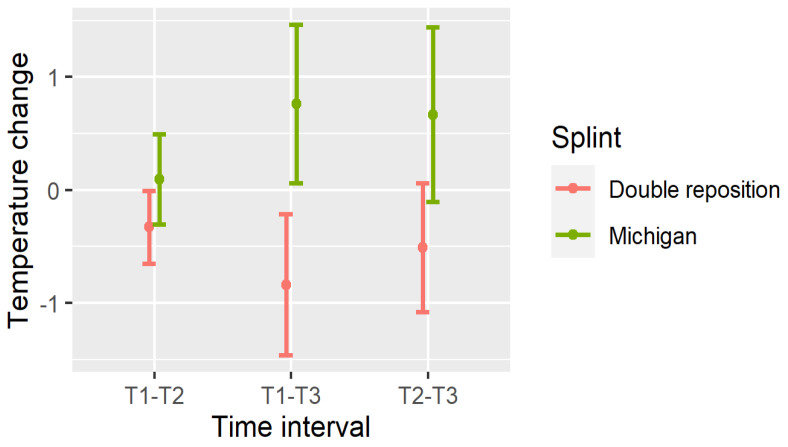
Temperature changes between measurements in N.

**Figure 8:**
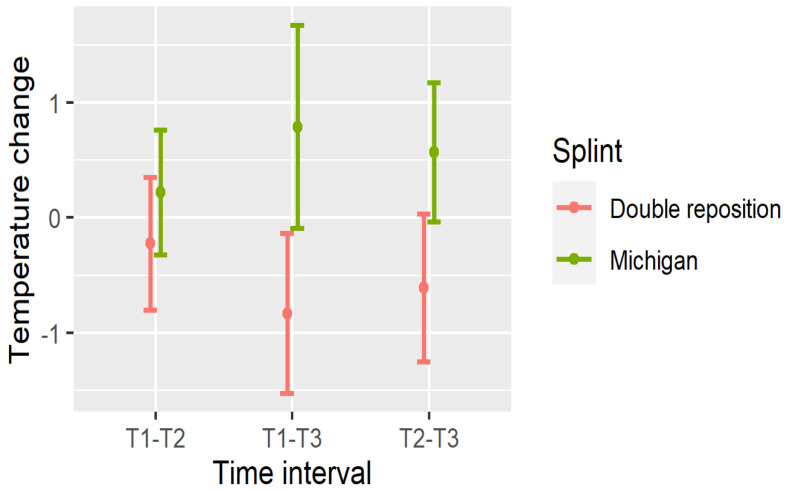
Temperature changes between measurements in OSP.

**Figure 9:**
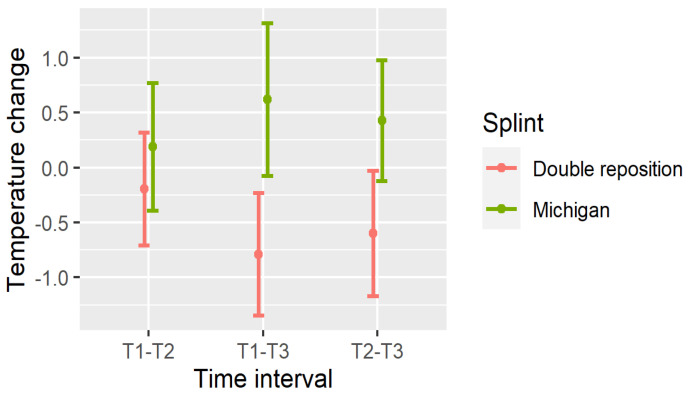
Temperature changes between measurements in OSL.

The significance of these temperature changes is summarized in Table 3, and visualized in Figure 7, Figure 8 and Figure 9.

The temperature changes between measurements are shown in Figure 7, Figure 8 and Figure 9.

In the double repositioning splint group (D), statistically significant temperature decreases were observed in nearly all comparisons:

- Masseter (DN): All intervals (T1–T2, T1–T3, T2–T3) showed significant reductions (*p* < 0.001);
- Suprahyoid (DOSP): Significant reductions were seen in T1–T3 and T2–T3, with a non-significant trend in T1–T2;
- Sternocleidomastoid (DOSL): There was a similar pattern, with the greatest drop observed in T1–T3 (*p* < 0.001).

In the Michigan splint group (M), temperature increased significantly in the masseter and suprahyoid regions between T1 and T3, and between T2 and T3 (*p* < 0.001). However, early changes (T1–T2) were not statistically significant, particularly for the suprahyoid and sternocleidomastoid muscles.

These findings indicate that the double repositioning splint induced a rapid and sustained reduction in surface muscle temperature, while the Michigan splint was associated with a gradual increase, particularly over the 30 min interval post-application.

Importantly, temperature reduction may reflect muscle relaxation and decreased metabolic activity, which supports the clinical effectiveness of the double repositioning splint in managing TMD-related muscle tension.

## 5. Comparison of the Splints

The differences in mean temperature and temperature changes between the two treatment groups are presented in Table 4.

At baseline (T1), no statistically significant differences in muscle temperature were observed between groups at any of the three anatomical sites, confirming comparable initial conditions. However, at T2, a significant difference emerged at the sternocleidomastoid muscle (OSL), and by T3, temperature values were significantly higher in the Michigan splint group (M) compared to the double repositioning splint group (D) across all muscle sites.

Regarding the direction of temperature change, the following were found:

- In group D (double repositioning splint), temperature consistently decreased over time at all measured locations.
- In group M (Michigan splint), temperature consistently increased, particularly between T1 and T3.

These trends were statistically significant for all time intervals and muscle sites, with *p*-values < 0.001 in nearly all comparisons. From a clinical perspective, the significant temperature reduction observed in group D supports its superior ability to relieve muscle tension, likely reflecting improved muscle relaxation and decreased metabolic activity. This effect is not only measurable via thermography but also palpable to clinicians during clinical examination, and is often reported by patients as subjective relief. By contrast, the temperature increase in group M may reflect persistent or increased muscle activation, suggesting a less immediate effect on reducing muscle tension.

## 6. Clinical Significance of Temperature Changes

In this study, a reduction in muscle surface temperature of approximately 0.8 °C was observed in the group treated with the double repositioning splint between T1 and T3. From a clinical standpoint, this change is meaningful as it may reflect a decrease in muscle activity and vascular perfusion, both of which are commonly associated with muscle relaxation. Elevated temperatures in superficial muscles are often linked to increased metabolic demand, inflammation, or sustained muscular contraction. Conversely, a drop in temperature suggests reduced neuromuscular excitation, lower energy expenditure, and potentially a decline in myofascial tension. These thermal shifts are consistent with findings in prior studies that used thermography to monitor the efficacy of interventions targeting hyperactive or overloaded muscles.

Although thermography alone cannot determine the exact levels of muscle tone, the observed temperature decrease exceeding 0.5 °C, as confirmed in multiple anatomical regions, can be considered clinically relevant, particularly when accompanied by subjective reports of symptom relief or improved range of motion. Therefore, the temperature reduction recorded in the double splint group may serve as a non-invasive biomarker of therapeutic muscle relaxation, supporting its use as an adjunctive measure in TMD management.

## 7. Discussion

Infrared thermography is a reliable, non-invasive, and radiation-free diagnostic method that facilitates the screening, evaluation, and monitoring of various clinical conditions. In the context of head and neck assessment, it enables the detection of thermal asymmetries that may indicate muscle hyperactivity or dysfunction [38]. Myofascial pain syndrome (MPS) is present in approximately 50% of temporomandibular disorder (TMD) cases and is typically diagnosed through physical examination, particularly palpation, as confirmed by Manfredini et al. [39]. Over the years, thermography has gained increasing recognition as a diagnostic tool in TMD, as demonstrated by Costa et al., Rodrigues-Bigaton et al., and Haddad et al., who have contributed to the standardization of thermographic image evaluation [40,41,42]. In our study, thermography was used not only for diagnostic purposes but also to compare the effects of two types of occlusal splints. The results demonstrated that the double repositioning splint produced a measurable decrease in muscle surface temperature, which may have indicated a reduction in muscle tone. Based on these findings, we rejected the null hypothesis in favor of the alternative, which proposed that different splint designs would result in distinct temperature profiles. This method was painless, non-contact, and well tolerated by all participants. To reduce variability, we recruited a homogeneous group of young European adults and standardized all environmental and procedural factors, including room temperature and camera positioning. The study design also addressed concerns raised in previous research. For example, Magalhães et al. reported that palpation may temporarily alter skin temperature. To avoid this confounding factor, all thermographic measurements were conducted at least one week after the initial clinical examination [43]. Our findings are consistent with earlier studies showing that occlusal splints can reduce muscle activity and inflammation. Treatment effectiveness appears to be influenced by splint design. The double repositioning splint may be particularly effective due to its ability to temporarily restrict mandibular movement, promoting muscle relaxation. Occlusal splints remain the most commonly used conservative approach for managing TMDs, as supported by the meta-analysis by Zhang et al., which demonstrated that splints can reduce pain intensity, increase maximum mouth opening, and lower muscle tension [44]. Through thermographic assessment, we were able to observe temperature reductions in specific anatomical structures, likely corresponding to decreased inflammation, as previously reported by Clemente et al. [45]. Conversely, the increase in temperature observed in the Michigan splint group may reflect continued muscular activation or insufficient relaxation, indicating a less immediate therapeutic effect compared to the double repositioning splint. While thermography holds promise as an objective evaluation method, it should be viewed as being complementary to, not a substitute for, comprehensive clinical examination. As Woźniak et al. noted, temperature changes are not specific to TMDs and may be influenced by other conditions [46]. Barbosa et al. also emphasized that infrared thermography did not significantly aid in differentiating TMD subtypes in their study, due to a lack of consistent correlation between thermal asymmetries and reported pain [47]. These limitations highlight the importance of applying standardized protocols, controlled environments, and calibrated equipment to obtain reliable thermographic results. Pain-related asymmetry is often associated with decreased muscle force on the more painful side, reflecting a compensatory pattern common in musculoskeletal disorders. Thermography has also been applied in specific populations, such as professional musicians. For example, in cases involving a violinist and a clarinetist, thermal imaging revealed changes in muscle activity patterns during occlusal splint therapy [48]. The successful treatment of TMDs should lead to a reduction in inflammation, which may be reflected by changes in surface temperature. However, the potential influence of concomitant medication use, including nonsteroidal anti-inflammatory drugs (NSAIDs) or corticosteroids, must be considered, as these substances may temporarily suppress inflammation and alter thermographic readings [49]. Barão et al. compared the therapeutic effects of occlusal splints and low-level laser therapy in TMD patients and found both methods reduced MPS symptoms, as evidenced by thermographic analysis [50]. Similarly, de Melo et al. emphasized the usefulness of thermography in the long-term monitoring of treatment outcomes. Importantly, thermal imaging may help identify hyperactive muscle regions and guide individualized treatment planning, including orthodontic or physiotherapeutic interventions [51]. Dibai-Filho et al. found a correlation between the severity of a TMD and a higher skin temperature over the TMJ, masseter, and anterior temporalis muscles [52]. In contrast, Haddad et al., in a study on female participants with myogenous TMD, observed lower skin temperatures in the masseter and temporalis muscles compared to healthy controls [53]. These inconsistent findings suggest that temperature response patterns may vary by TMD subtype and demographic factors. The authors noted that their sample size was limited and recommended further studies in larger populations to validate their results.

## 8. Conclusions

1. Thermography using a thermal imaging camera is a precise, objective, and non-invasive diagnostic method that can be effectively applied to assess masticatory muscle tone in patients with temporomandibular disorders (TMDs).
2. The type of occlusal splint significantly influences thermal patterns and, consequently, muscle activity.
3. The double repositioning splint showed greater efficacy in reducing surface muscle temperature, suggesting its potential for short-term relief in patients with muscular or myofascial TMD.
4. Further research should be conducted on larger and more diverse populations to confirm these findings and assess their generalizability across different age groups, sexes, and ethnic backgrounds.

## Figures and Tables

**Table 1:** Inclusion and exclusion criteria.

Inclusion Criteria	Exclusion Criteria
Pain in the tissues surrounding the temporomandibular joint (TMJ)	Absence of pain in the TMJ region
Consent to avoid hot drinks and spicy foods	Lack of consent to follow dietary restrictions
Consent to avoid hot baths or microcirculation-stimulating activities	Lack of consent to avoid heat or circulatory stimulation
Age between 20 and 25 years	Age outside the 20–25 range
European origin (Polish ethnicity)	Non-European origin

**Table 2:** Descriptive statistics for temperature in the double repositioning splint group (D) and the Michigan splint group (M).

	Mean ± SD	[Min, Max]	95% Conf. Interval
group (D), double repositioning splint			
N, T_1_	35.09 ± 1.24	[31.8, 36.7]	(34.62, 35.55)
N, T_2_	34.76 ± 1.19	[31.5, 36.4]	(34.31, 35.2)
N, T_3_	34.25 ± 1.48	[30.1, 36.2]	(33.69, 34.8)
OSP, T_1_	35.05 ± 1.11	[32, 36.6]	(34.64, 35.47)
OSP, T_2_	34.83 ± 1.08	[31.9, 36.5]	(34.43, 35.23)
OSP, T_3_	34.22 ± 1.34	[30.6, 36.4]	(33.72, 34.72)
OSL, T_1_	34.93 ± 1.15	[32.1, 36.3]	(34.5, 35.36)
OSL, T_2_	34.74 ± 1.10	[31.6, 36.2]	(34.33, 35.15)
OSL, T_3_	34.14 ± 1.28	[31.1, 36.1]	(33.66, 34.62)
group (M), Michigan splint			
N, T_1_	34.92 ± 1.16	[32.1, 36.7]	(34.49, 35.36)
N, T_2_	35.02 ± 1.13	[32.4, 36.9]	(34.6, 35.44)
N, T_3_	35.69 ± 1.04	[33, 37.2]	(35.3, 36.07)
OSP, T_1_	35.16 ± 1.27	[31.6, 36.9]	(34.69, 35.64)
OSP, T_2_	35.38 ± 1.13	[33.1, 37.2]	(34.96, 35.8)
OSP, T_3_	35.95 ± 0.95	[33.5, 37.8]	(35.6, 36.31)
OSL, T_1_	35.34 ± 1.15	[32.6, 36.8]	(34.91, 35.77)
OSL, T_2_	35.53 ± 0.93	[33.2, 36.9]	(35.18, 35.87)
OSL, T_3_	35.96 ± 0.81	[34.3, 37.2]	(35.65, 36.26)

**Table 3:** The significance of the temperature changes at different time points.

	Mean	SD	*p*-Value	95% CI
group (D), double repositioning splint				
DN_12_	−0.33	0.323	<0.001	(−0.451, −0.209)
DN_13_	−0.84	0.625	<0.001	(−1.073, −0.607)
DN_23_	−0.51	0.571	<0.001	(−0.723, −0.297)
DOSP_12_	−0.223	0.578	0.129	(−0.439, −0.008)
DOSP_13_	−0.83	0.695	<0.001	(−1.089, −0.571)
DOSP_23_	−0.607	0.642	<0.001	(−0.846, −0.367)
DOSL_12_	−0.193	0.514	0.145	(−0.385, −0.001)
DOSL_13_	−0.79	0.557	<0.001	(−0.998, −0.582)
DOSL_23_	−0.597	0.57	<0.001	(−0.810, −0.384)
group (M), Michigan splint				
DN_12_	0.095	0.398	0.603	(−0.054, 0.244)
DN_13_	0.763	0.701	<0.001	(0.501, 1.024)
DN_23_	0.668	0.772	<0.001	(0.379, 0.956)
DOSP_12_	0.22	0.542	0.103	(0.018, 0.422)
DOSP_13_	0.79	0.881	<0.001	(0.461, 1.119)
DOSP_23_	0.57	0.603	<0.001	(0.345, 0.795)
DOSL_12_	0.19	0.581	0.251	(−0.027, 0.407)
DOSL_13_	0.62	0.694	<0.001	(0.361, 0.880)
DOSL_23_	0.43	0.549	<0.001	(0.225, 0.635)

**Table 4:** Differences between the groups in mean temperature and temperature change.

	Group (D), Double Repositioning Splint			Group (M), Michigan Splint		
	Mean	SD		Mean	SD	*p*-Value
Temperature measurements						
N, T_1_	35.09	1.24		34.92	1.158	0.604
N, T_2_	34.76	1.187		35.02	1.132	0.383
N, T_3_	34.25	1.483		35.69	1.036	<0.001
OSP, T_1_	35.05	1.114		35.16	1.275	0.723
OSP, T_2_	34.83	1.079		35.38	1.128	0.057
OSP, T_3_	34.22	1.338		35.95	0.951	<0.001
OSL, T_1_	34.93	1.149		35.34	1.147	0.179
OSL, T_2_	34.74	1.099		35.53	0.927	0.004
OSL, T_3_	34.14	1.284		35.96	0.815	<0.001
Temperature change						
DN_12_	−0.33	0.323		0.095	0.398	<0.001
DN_13_	−0.84	0.625		0.76	0.701	<0.001
DN_23_	−0.51	0.571		0.67	0.772	<0.001
DOSP_12_	−0.223	0.578		0.22	0.542	0.009
DOSP_13_	−0.83	0.695		0.79	0.881	<0.001
DOSP_23_	−0.607	0.642		0.57	0.603	<0.001
DOSL_12_	−0.193	0.514		0.19	0.581	0.027
DOSL_13_	−0.79	0.557		0.62	0.694	<0.001
DOSL_23_	−0.597	0.570		0.43	0.549	<0.001

## Data Availability

The datasets used to support the findings of this study are available from the corresponding author upon request.
